# Trends of Negotiated Targeted Anticancer Medicines Use in China: An Interrupted Time Series Analysis

**DOI:** 10.34172/ijhpm.2021.47

**Published:** 2021-06-09

**Authors:** Cong Huang, Carolina Oi Lam Ung, Haishaerjiang Wushouer, Lin Bai, Xinyi Li, Xiaodong Guan, Luwen Shi

**Affiliations:** ^1^Department of Pharmacy Administration and Clinical Pharmacy, School of Pharmaceutical Sciences, Peking University, Beijing, China.; ^2^State Key Laboratory of Quality Research in Chinese Medicine, Institute of Chinese Medical Sciences, University of Macau, Macao, China.; ^3^Center for Strategic Studies, Chinese Academy of Engineering, Beijing, China.

**Keywords:** Price Negotiation, Targeted Anticancer Medicines, Interrupted Time Series, China

## Abstract

**Background:** In order to relieve the financial burden of the patients in China, the Ministry of Health (MoH) conducted the first national price negotiation and successfully negotiated three expensive medicines including 2 targeted anticancer medicines (TAMs), icotinib and gefitinib. However, little evidence was available to demonstrate the impact of the national negotiation on TAMs use. The purpose of the study is to evaluate the implementation of the national price negotiation policy in China on TAMs use.

**Methods:** We used interrupted time series (ITS) design to examine the changes in the daily cost, the monthly hospital purchasing volume and spending of icotinib and gefitinib with pharmaceutical procurement data from 594 tertiary hospitals in 29 provinces of mainland China between January 2015 and July 2017. The period between May and July 2016 was applied to assess the impact of policy.

**Results:** The daily cost of icotinib and gefitinib decreased by 50.08% (*P*<.001) and 53.89% (*P*<.001) 12 months after the national negotiation, respectively. In terms of volume, the negotiation was associated with increases in the trend of the monthly hospital purchasing volume of icotinib and gefitinib by 4.87 thousand defined daily doses (DDDs) (*P*<.001) and 6.89 thousand DDDs (*P*<.001). However, the monthly hospital purchasing spending of icotinib and gefitinib decreased rapidly by US$0.51 million (*P*<.010) and US$0.82 million (*P*<.050) following policy implementation, respectively.

**Conclusion:** The first national negotiation had successfully cut off the price of two negotiated TAMs and promoted TAMs use in China. In the future, government should conduct further price negotiations and include more medicines with clinical benefits into reimbursement schemes to alleviate patients’ financial burden and promote their access to essential treatment.

## Background

**Key Messages**

**Implications for policy makers**

- National price negotiation can cut off the price of negotiated targeted anticancer medicines (TAMs).
- Another implication was that price negotiation can help controlling pharmaceutical spending.
- To control increasing medicine expenditures, government should conduct further price negotiations for more medicines with clinical benefits.

**Implications for the public**

Evidences indicated that national price negotiation had successfully cut off the price of two negotiated targeted anticancer medicines (TAMs). Our findings also suggested that the national negotiation was successful at encouraging the use of TAMs, promoting the access and improving patients’ affordability.

###  High Medicine Expenditure

 According to the World Health Organization (WHO) report, the average national percentage of total government expenditure devoted to healthcare is 11.7%^1^ and measured total pharmaceutical expenditure accounts for 1.41% to 1.63% of gross domestic product (GDP).^2^ In China, the total annual expenditure of health had reached US$794 billion, accounting for 6.36% of GDP in 2018.^3^ In 2012, pharmaceutical expenditure accounted for more than 40% of all health expenditure and had grown faster than GDP of China since 1990.^4^ Reasonable factors for rapidly increasing expenditure on medicines include more people being treated and the diffusion of new drugs,^5^ especially the expensive medicines for serious illnesses. Chinese patients devoted 30%–40% of their out-of-pocket healthcare expenditures to medicines between 2010 and 2017,^3^ which brought heavy financial burden and subsequently led to financial barriers for access to medicines.^6^ Incorporating cost of medicines into the coverage of health insurance reimbursement would relieve the financial burden of patients and promote access.^7-9^

###  Health Insurance Schemes and National Medicine Negotiation in China

 In order to relieve the financial burden of Chinese patients, since October 2015, the Ministry of Health (MoH) started to employ national drug price negotiation.^10^ Three medicines of three pharmaceutical manufacturers were applied to enter the negotiation. MoH set to, upon successful negotiation, include the expensive medicines in the health insurance schemes and bulk purchase them with priority from 2016 to 2017. During the national negotiation, three basic social health insurance programs of the China’s social health insurance schemes were involved.^8^ New Rural Cooperative Medical Scheme was designed for the rural population, which covered 48.7% of the Chinese population in 2015.^11^ Urban Resident Basic Medical Insurance targeted the unemployed, the disabled, children and elderly people in urban areas. Urban Employed Basic Medical Insurance was designed for urban employees.^12^

 On May 20, 2016, the General Office of the State Council announced the results of the negotiation, and three successfully negotiated medicines had over 50% price reduction (Table 1).^13^ MoH requested: (1) the medicine centralized tender bidding system in each province updated the prices so that all the public hospitals could procure the medicines at the negotiated prices by the end of June 2016; and (2) all provincial health insurance administrations should list the three medicines in the reimbursement schemes.

**Table 1:** Descriptive Information of the Three Successfully Negotiated Medicines

**Generic Name**	**Brand Name**	**Approval date in China**	**Marketing Authorization Holder**	**DDD** ^a^ **(mg)**	**Therapeutic Class** ^b^	**ATC Code**	**Negotiated Daily Cost** ^c^ ** (USD** ^d^)
Tenofovir disoproxil fumarate	Viread	6/18/2008	Gilead Sciences Inc.	300	HIV-1; HBV	J05AF07	2.46
Icotinib	Conmana	6/7/2011	Bettapharma Inc.	375	Non-small-cell lung cancer with somatic EGFR mutations	L01XE48	30.09
Gefitinib	IRESSA	12/6/2004	AstraZeneca AB	250	Non-small-cell lung cancer with somatic EGFR mutations	L01XE02	35.50

Abbreviations: DDD, defined daily dose; ATC, anatomical therapeutic chemical; EGFR, epidermal growth factor receptor; HBV, hepatitis B virus. ^a^DDD of tenofovir disoproxil fumarate was the daily amounts based on dosage regimen recommended by WHO.^14^ As for icotinib and gefitinib, we calculated DDD for each product in consideration of the dosage regimen recommended in the manufacturers’ instructions of products, as approved by National Medical Products Administration.^15^ ^b^ Therapeutic Class: Summarized from indications in the manufacturers’ instructions of products approved by National Medical Products Administration. ^c^ Negotiated daily cost: calculated in terms of DDD and negotiated prices. ^d^ USD: US dollar based on the May 2016 exchange rate.^16^

###  Lung Cancer and High Disease Burden

 Lung cancer was the malignant tumor with the highest incidence and mortality in Chinese population.^17^ Related studies showed that the lifetime cost of drugs associated with lung cancer treatment was US$10 664.05, which would lead to serious financial burden to the majority of patients in China.^18^ In the successfully price-negotiated medicines, both icotinib and gefitinib were the first-line treatment for non-small-cell lung cancer with somatic epidermal growth factor receptor (EGFR) mutations in China.^19,20^ Before the negotiation, patients prescribed icotinib had to pay over US$2000 every month while Chinese annual per capita disposable income was US$3532.2 in the same period.^21^ Serious financial burden restricted patients’ access to targeted anticancer medicines (TAMs). A multicenter survey in China found that only two thirds of EGFR positive patients with unresectable Stage IIIB/IV nonsquamous non-small cell lung cancer received tyrosine kinase inhibitors treatment.^22^

 Scientific evidence on medicine use is essential for government reimbursement policy design and evaluation.^23^ To our knowledge, there is no previous study providing evidence on TAMs utilization of the first national negotiation in China. The aim of our study was to examine the differences in the daily cost, the hospital purchasing volume and spending of icotinib and gefitinib pre- and post-enlistment.

## Methods

###  Study Design

 We used interrupted time series (ITS)^24^ design with hospital procurement data to analyze the policy effect on negotiated TAMs, icotinib and gefitinib, from January 2015 to July 2017. For the purpose of sensitivity analysis and taking into account the possible lagged effect of policy, we selected May 2016 to July 2016 as “phase-in” period according to the date of negotiation results announcement made by the MoH on May 20, 2016.^25^

###  Data Sources

 We used the data from China Medicine Economic Information, a large database covering procurement records of public hospitals in mainland China.^26^ We extracted data of icotinib and gefitinib purchased by 594 tertiary hospitals from 29 provinces of mainland China (Qinghai and Gansu not included) between January 2015 to July 2017. Aggregated procurement data included the monthly purchasing volume and spending of the TAMs selected in this study, as well as their dosage form, strength, purchase time, the Anatomical Therapeutic Chemical code^27^ and manufacturer. The study was considered not human subjects research by the Peking University Health Science Review Board.

###  Outcome Measures

 We applied three main outcome measures: the daily cost, the hospital purchasing volume (numbers of defined daily dose, DDD) and spending for each of icotinib and gefitinib. The hospital purchasing volume and spending was the sum of purchasing volume and spending of each hospital. The DDD referred to the daily amounts based on dosage regimen recommended in the manufacturers’ instructions as approved by National Medical Products Administration.^15^ The daily cost (cost of DDD) was calculated as follows:


$$Daily\;\cos t=\frac{Hospital\;purcha\sin g\;spending}{Hospital\;purcha\sin g\;volume\left(DDDs\right)}$$

All expense data were reported in US dollar (US$1 = CNY 6.2284 based on the 2015 exchange rate^28^) after adjusted to January 2015 using the medical care component of the Consumer Price Index.^29^

###  Statistical Analysis

 We conducted seasonal adjustment and assessed the outcomes overtime for the 2 negotiated TAMs, icotinib and gefitinib.^30^ We used segmented regression model to estimate changes in the daily cost, the hospital purchasing volume and spending. The regression equation is as follows:


$$Y_{t}=\beta_{0}+\beta_{1}*time_{t}+\beta_{2}*level_{t}+\beta_{3}*trend_{t}+\varepsilon_{t}$$

*Y*_t_ represented the daily cost, the hospital purchasing volume and spending at time *t*. *β*_0_ was a constant term, which estimated the baseline level of *Y*_t_. *β*_1_ estimated the trend of *Y*_t_ prior to the policy. *β*_2_ estimated the level change of the outcomes immediately following the policy. *β*_3_ estimated the trend change after the intervention. *ε*_t_ represented the random error at time *t. *We presented changes in the level or trend of the daily cost, the hospital purchasing volume and spending. To estimate the combined change of level and trend, we calculated the absolute and relative differences (with 95% CIs) at 12 months after policy compared to the estimated *Y*_t_ had the intervention not happened.^31^

 The Durbin-Watson statistic was used to test a serial autocorrelation of the error terms in the regression model.^32^ We used the Cochrane-Orcutt auto-regression procedure to correct first order serially correlated errors when needed.^33^ All statistical analysis was performed on STATA/SE V.15.0. except for the seasonal adjustment on EVIEWS 10.

## Results

###  ITS Analysis of Changes in the Daily Cost of Icotinib and Gefitinib

**Figure 1:**
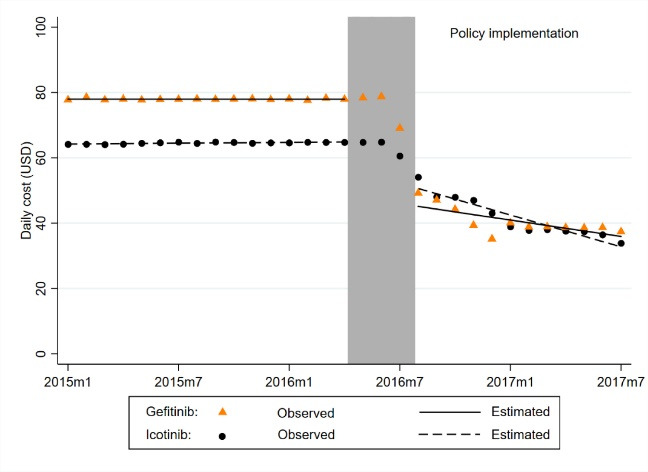
Changes in the Daily Cost for Icotinib and Gefitinib, 2015-2017.

 The daily cost of both icotinib and gefitinib declined over time after the implementation of the policy (Figure 1, Table 2). Before June 2016, the daily cost of icotinib was stable at about US$64.22 and gefitinib was about US$77.96. After the national negotiation, the daily cost of icotinib experienced a significant level decrease of US$14.26 (*P* < .001) while the daily cost of gefitinib decreased by US$32.83 (*P* < .001) in August 2016. By the end of observation (12 months after implementation), the daily cost of icotinib and gefitinib was 50.08% (*P* < .001) and 53.89% (*P* < .001) lower than what would have been expected in the absence of the policy respectively.

**Table 2:** Estimates From Interrupted Time-Series Models of the Impacts of National Negotiation on the Daily Cost, the Monthly Hospital Purchasing Volume and the Monthly Hospital Purchasing Spending for Icotinib and Gefitinib

**Variable**	**Daily Cost (USD)**		**Hospital Purchasing Volume (Thousand DDD)**		**Hospital Purchasing Spending (Million USD)**	
	**β**	**95% Cl**	**β**	**95% Cl**	**β**	**95%Cl**
**Icotinib**						
Baseline level	64.22^c^	64.02 to 64.42	40.41^c^	38.27 to 42.56	2.58^c^	2.44 to 2.72
Baseline trend	0.04^c^	0.02 to 0.06	0.89^c^	0.66 to 1.12	0.06^c^	0.05 to 0.08
Level change	-14.26^c^	-16.87 to -11.66	0.81	-5.50 to 7.12	-0.51^b^	-0.83 to -0.19
Trend change	-1.66^c^	-2.04 to -1.29	4.87^c^	4.00 to 5.74	0.02	-0.03 to 0.07
Absolute change	-32.57^c^	-35.12 to -30.02	54.34^c^	47.58 to 61.10	-0.26	-0.68 to 0.15
Relative change (%)	-50.08^c^	-53.80 to -46.36	93.27^c^	79.24 to 107.31	-6.94	-17.11 to 3.23
**Gefitinib**						
Baseline level	77.96^c^	77.73 to 78.20	53.24^c^	48.64 to 57.84	4.12^c^	3.72 to 4.51
Baseline trend	0.00	-0.02 to 0.02	0.42	-0.08 to 0.92	0.04	0.00 to 0.07
Level change	-32.83^c^	-37.74 to -27.91	23.23^c^	13.09 to 33.37	-0.82^a^	-1.53 to -0.11
Trend change	-0.83^b^	-1.43 to -0.24	6.89^c^	5.68 to 8.10	0.16^c^	0.08 to 0.24
Absolute change	-42.01^c^	-44.64 to -39.38	99.05^c^	86.93 to 111.18	0.92^a^	0.08 to 1.77
Relative change (%)	-53.89^c^	-57.07 to -50.72	160.50^c^	126.76 to 194.25	19.16^a^	0.97 to 37.36

Abbreviation: DDD, defined daily dose. ^a^ *P *<.050; ^b^*P *<.010; ^c^*P *<.001. Absolute change, Relative change: Change at 12 months after policy implementation.

###  ITS Analysis of Changes in the Hospital Purchasing Volume and Spending of Icotinib and Gefitinib

**Figure 2:**
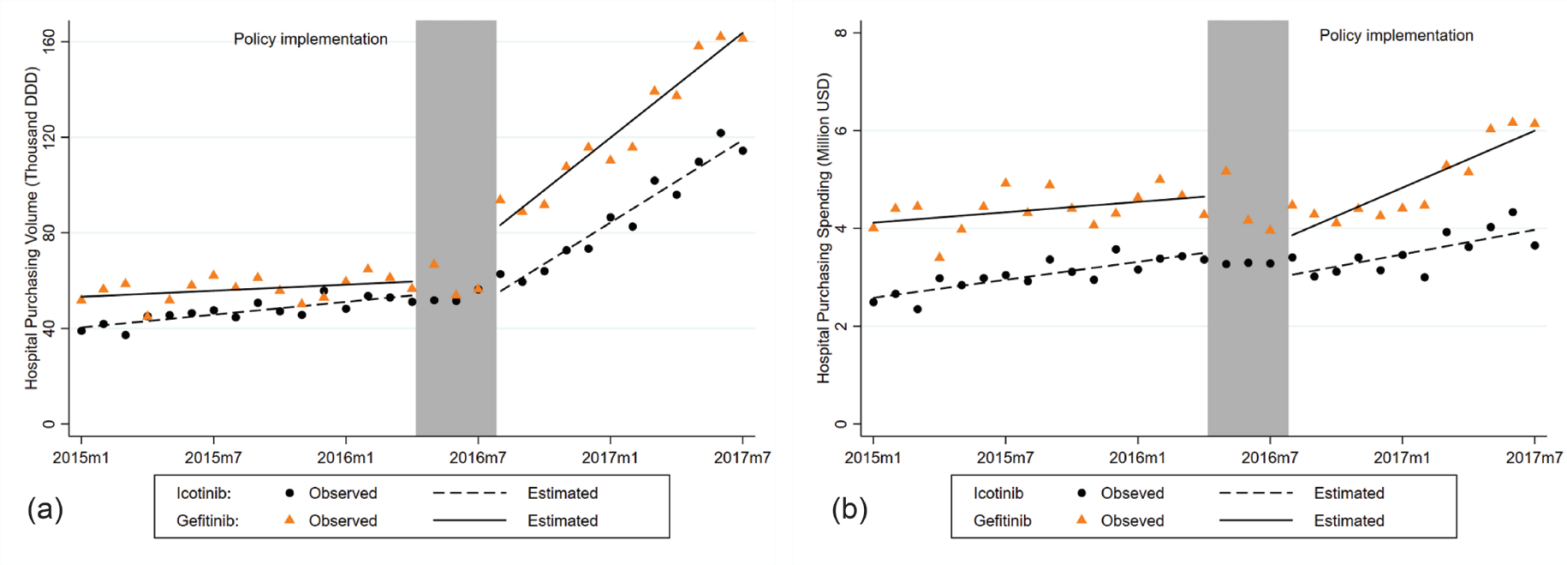
Changes in the Hospital Purchasing Volume Per Month (a) and the Hospital Purchasing Spending Per Month (b) for Icotinib and Gefitinib, 2015-2017.

 The purchasing volume of icotinib and gefitinib increased significantly after implementation of the policy, while the level of purchasing spending decreased significantly (Figure 2, Table 2). The purchasing volume of icotinib per month was increasing by 0.89 thousand DDDs (*P* < .001) prior to the policy. Following the national negotiation policy, the purchasing volume of icotinib increased by 4.87 thousand DDDs (*P* < .001) in the trend. By the end of observation, the purchasing volume increased by 93.27% (*P* < .001). The purchasing spending of icotinib per month was increasing by US$0.06 million (*P* < .001) prior to the policy. However, after policy implementation, there was a decrease of US$0.51 million (*P* < .010) in the level, but no significant change at 12 months after policy. Similarly for gefitinib, the implementation of national negotiation policy was associated with a significant increase of 6.89 thousand DDDs (*P* < .001) in the trend and 23.23 thousand DDDs (*P* < .001) in the level of purchasing volume, resulting in an estimated increase of 160.50% (*P* < .001) in the end. The purchasing spending of gefitinib decreased by US$0.82 million (*P* < .050) in the level and increased by US$0.16 million (*P* < .001) in the trend, resulting in an estimated increase of 19.16% (*P* < .050) in the last month of the observation period.

## Discussion

 We found that China’s first national negotiation had successfully decreased the daily cost of two negotiated TAMs, and the hospital purchasing volume of both icotinib and gefitinib increased significantly. However, the level of hospital purchasing spending of two TAMs decreased rapidly following policy implementation. These findings suggested that the national negotiation was successful at encouraging the use of anticancer medications, promoting the access and improving patients’ affordability.

 Cancer has been the leading cause of death in China with increasing incidence and mortality,^34,35^ which has caused a tremendous economic burden on Chinese patients.^36,37^ TAMs have been the focus of cancer drug development for the past two decades,^38,39^ which in the meanwhile raised major concerns over their affordability due to high prices.^40,41^ We found that the first national medicine negotiation in China slashed the average daily cost of icotinib and gefitinib by over 50%. Another two negotiations conducted in 2017 and 2018 involving 48 medicines (including 30 TAMs^42,43^) also saw a price cut of more than 40%.^44^ The negotiation policy effectively lowered drug prices providing the beneficiaries of the insurance schemes better opportunities to get more affordable access to lifesaving yet expensive TAMs. However, in the US, the expected cut in the prices of anticancer drugs did not happen post the launch of the negotiation policy in many countries, instead, an increase in drug price was observed,^45^ especially for TAMs.^46^ While the Medicare and Medicaid of the United States government were still deciding whether or not to fully implement the drug price negotiation system,^47-49^ our study evidence showed that the drug price negotiation policy in China had successfully achieved medicine price control.

 The positive impact of the national negotiation on utilization was also observed in this study. The significant increase in the hospital purchasing volume for icotinib (93.27%) and gefitinib (160.50%) 12 months after the implementation of the policy, indicating that national negotiation had possibly led to better access to these expensive medications due to improved affordability. Other studies analyzing provincial negotiation in China showed similar positive effects on utilization and affordability of expensive TAMs.^23,50^ As recommended by the WHO, tendering and negotiation are pricing approaches for determining the price that is mutually agreeable for both the sellers and the buyers, which have been used for establishing the price of cancer medicines by some authorities.^51^ Apart from China, the establishment of the negotiating commission seems to have led to reduced prices and possibly better access in other middle-income countries.^52^ In developed countries like Germany, price negotiations led to a 24.5% decrease in negotiated prices relative to launch prices.^53^ Consistent with these previous findings, price reduction together with enlistment on the health insurance reimbursement scheme was important for promoting utilization of anticancer medicines.^23,54,55^

 Another implication was that price negotiation could help controlling pharmaceutical spending. At hospital level, the purchasing spending of both icotinib and gefitinib decreased rapidly following policy implementation. The short-term effects might result from the price reduction of over 50%. In the long term, the purchasing spending of icotinib showed no significant change while the purchasing spending of gefitinib increased by about 19.16% one year after implementation of policy. However, the increase was based on the explosion of purchasing volume of 160.50%. In other words, the price negotiation can help control pharmaceutical spending, together with promoting medicine utilization. However, the monitoring of negotiated medicines utilization was needed to refrain from irrational use and control increasing expenditures. However, it is important to note that the impact of fragmentation in social health insurance schemes might have on the price outcome of the policy for the patients. Across different provinces in China, negotiated medicines were enlisted in various health insurance schemes according to the local situations, and thus people were subject to different levels of financial protection covered by the health insurance schemes.^8^ Collectively, it might worsen the overall inequity in terms of accessibility of TAMs affecting the health and quality of life for some patients.^56^ Furthermore, even though the costs of the two TAMs drastically declined by over 50% after negotiation as shown in this study, the affordability at patient level still posed serious problem for the low-income population, especially those from Western regions.^23^ Other supplementary measures such as catastrophic medical insurance and medical aid should be in place to top up the basic cover offered by the basic social health insurance schemes and provided extra financial protection to vulnerable groups and needy populations.^57^

###  Limitations

 There were several limitations in the study. Firstly, due to limited data access, other TAMs were not included in this study which could be used as control group to further strengthen the study design and to reduce, estimated bias to the study findings. Secondly, only around 30% of all tertiary hospitals in China were included in our study,^11^ but we believed that the study findings would still be sufficiently representative because the 594 tertiary hospitals included in this study were from 29 provinces of mainland China (except Qinghai and Gansu). Thirdly, secondary hospitals and pharmacies where icotinib and gefitinib might be assessed were not enrolled in this study. However, the impact was expected to be minimal as TAMs were mostly assessed through tertiary hospitals in China. Cancer patients in China usually prefer to seek medical care in tertiary hospitals due to the severity of the disease and the inadequate healthcare resources in the primary health institutes. Fourthly, the influence of related patient assistance programs was not considered in the analysis. Such programs like the Iressa Means–Tested Drug Donation offered free Iressa (gefitinib) to low-income patients who could not afford continuous treatment after they paid for the first several courses of treatment.^58^ However, the number of patients who participated in patient assistance programs was limited,^59^ and little influence was expected on the estimated results in this study. Lastly, the differences in the implementation time and reimbursement schemes among targeted provinces might have led to bias. With aggregated procurement data, we could not distinguish medicine prescribed for patients covered by different reimbursement schemes. Also, we also could not directly assess patients’ access to medicines due to the limitation of procurement data used in this study. Further study with claim data or prescription data is needed to close the research gap.

## Conclusion

 The national negotiation policy had successfully cut off the price of two negotiated medicines and promoted their use in China. After implementation of the policy, the purchasing volume of icotinib and gefitinib increased significantly at hospital level. The decrease of daily cost indicated that the out-of-pocket expenses by the patients were reduced. However, further study with claim data or prescription data is needed to provide more evidence on how patients’ access to different TAMs changes with national negotiation. In the future, it is important for the Chinese government to continuously carry out the price negotiations and include more TAMs with clinical benefit into reimbursement schemes in order to alleviate patients’ financial burden and promote access to their essential treatment.

## Acknowledgements

 All authors are also grateful to staff of Chinese Pharmaceutical Association for their support and cooperation in data access and analysis. The contents are solely the responsibility of the authors, and do not reflect the views of the funding bodies or any organization.

## Ethical issues

 We used secondary data from Chinese Medical Economic Information database. As such, ethical approval was not required.

## Competing interests

 Authors declare that they have no competing interests.

## Authors’ contributions

 LS and XG conceptualised and designed the study. CH and LB contributed to analysis of the data. XG, CH, HW, and XL conducted the final analyses. COLU and CH drafted the initial manuscript. All authors contributed to the critical revision of the manuscript and approved the final version.

## Funding

 This work was supported by National Natural Science Foundation of China (Grant No.71774005). The funders had no role in study design, data collection and analysis, decision to publish, or preparation of the manuscript.
